# An Adult Thymic Stromal-Cell Suspension Model for *in Vitro* Positive Selection

**DOI:** 10.1155/1998/10534

**Published:** 1998

**Authors:** Ann P. Chidgey, Hanspeter Pircher, H. Robson Macdonald, Richard L. Boyd

**Affiliations:** 1 Department of Pathology and Immunology Monash Medical School Commercial Rd. Prahran 3181 Australia; 2 Department of Immunology Institute for Medical Microbiology and Hygiene University of Freiburg Germany; 3 Ludwig Institute for Cancer Research Epalinges 1066 Switzerland

**Keywords:** Positive selection, T-cell differentiation, thymic selection, LCMV, peptides

## Abstract

Presented here is a cell-suspension model for positive selection using thymocytes from *αβ*-TCR (H-2D^b^-restricted) transgenic mice specific to the lymphocytic choriomeningitis virus (LCMV) on a nonselecting MHC background (H-2^d^ or TAP-1 –/–), cocultured with freshly isolated adult thymus stromal cells of the selecting MHC type. The thymic stromal cells alone induced positive selection of functional CD4^-^CD8^+^ cells whose kinetics and efficiency were enhanced by nominal peptide. Fibroblasts expressing the selecting MHC alone did not induce positive selection; however, together with nonselecting stroma and nominal peptide, there was inefficient positive. These results suggest multiple signaling in positive selection with selection events able to occur on multiple-cell types. The ease with which this model can be manipulated should greatly facilitate the resolution of the mechanisms of positive selection in normal and pathological states.


*Developmental Immunology*, 1998, Vol. 6, pp. 157–170
Reprints available directly from the publisher
Photocopying permitted by license only

© 1998 OPA (Overseas Publishers Association)
N.V. Published by license under the
Harwood Academic Publishers imprint,
part of the Gordon and Breach Publishing Group
Printed in Malaysia

### An Adult Thymic Stromal-Cell Suspension Model for *In Vitro* Positive Selection

ANN P. CHIDGEY[a]* HANSPETER PIRCHER[b] H. ROBSON MACDONALD[c] RICHARD L. BOYD[a]

[a]*Department of Pathology and Immunology, Monash Medical School, Commercial Rd., Prahran 3181, Australia*; [b]*Department of Immunology, Institute for Medical Microbiology and Hygiene, University of Freiburg, Germany*; [c]*Ludwig Institute for Cancer Research, 1066 Epalinges, Switzerland*

*(Received 20 January 1997; Accepted 19 August 1997)*

Presented here is a cell-suspension model for positive selection using thymocytes from $\alpha\beta$-TCR (H-2D$^b$-restricted) transgenic mice specific to the lymphocytic choriomeningitis virus (LCMV) on a nonselecting MHC background (H-2$^d$ or TAP-1 $-/-$), cocultured with freshly isolated adult thymus stromal cells of the selecting MHC type. The thymic stromal cells alone induced positive selection of functional CD4$^-$CD8$^+$ cells whose kinetics and efficiency were enhanced by nominal peptide. Fibroblasts expressing the selecting MHC alone did not induce positive selection; however, together with nonselecting stroma and nominal peptide, there was inefficient positive. These results suggest multiple signaling in positive selection with selection events able to occur on multiple-cell types. The ease with which this model can be manipulated should greatly facilitate the resolution of the mechanisms of positive selection in normal and pathological states.

*Keywords:* Positive selection, T-cell differentiation, thymic selection, LCMV, peptides

### INTRODUCTION

Engagement of the TCR is a pivotal step in thymocyte development, ultimately resulting in the survival (positive selection) or loss (negative selection) of developing T cells. The molecular and cellular interactions necessary for these selection events, however, are still poorly understood. MHC transfected fibroblast lines (Pawlowski et al., 1993) and cloned thymic epithelial-cell lines (Hugo et al., 1992; Vukmanovic et al., 1992) were able to induce positive selection after intrathymic injection. However, the possibility for multicellular involvement—fibroblasts providing the selecting MHC combined with appropriate thymic epithelial cells delivering inducing signals—has not been addressed.

In conjunction with TCR transgenic mice, fetal thymic organ culture (FTOC) has been a useful *in vitro* model for investigating the effects of peptide in thymic selection processes (Ashton-Rickardt et al., 1994; Hogquist et al., 1994; Sebzda et al., 1994). Reaggregate FTOC have also been developed to

*Corresponding author.

include defined populations of T-cell precursors and embryonic stroma (Anderson et al., 1994; Merkenschlager and Fisher, 1994). However, in these FTOC models, embryonic thymi are the source of T-cell precursors and stroma, yet the nature of the embryonic thymus differs markedly from that of the adult (Boyd et al., 1993). Hence, they may be inappropriate to study the mechanisms and cellular involvement in adult thymic positive selection and abnormalities therein that are manifest postnatally. Furthermore, the downregulation of RAG-1 expression in immature $CD4^+CD8^+$ thymocytes (Brandle et al., 1992; Kouskoff et al., 1995), a common source of precursor cells for some current assays for positive selection, indicates that cells at this stage may have already received selection signals *in vivo*, prior to culture. We thus sought to develop an *in vitro* model for positive selection that uses adult stroma and requires minimal pretreatment of T-cell precursors yet eliminates the possibility that selection signals had already occurred *in vivo*.

This cell-suspension model is more easily manipulated and should enable a more detailed dissection of the thymic stromal elements involved in adult thymic selection and hence allow investigations into possible thymic selection defects present in autoimmune diseases.

### RESULTS

### An Adult Thymic Stromal-Cell-Suspension Model for *In Vitro* Positive Selection

The basic method is outlined in Figure 1. P14 transgenic mice expressing a TCR ($V\beta8.1$, $V\alpha2$) specific for the LCMV glycoprotein peptide (amino acid sequence 33-41; KAVYNFATM), restricted to $H\text{-}2D^b$, were bred onto a nonselecting MHC ($H\text{-}2^d$) background. Rag-1 expression is not downregulated in TCR transgenic $CD4^+CD8^+$ thymocytes on a nonselecting MHC background, suggesting that positive selection signals had not occurred (Kouskoff et al., 1995). Day 17-18 gestation was optimal for target cells since we found this was the earliest age with significant levels of $CD4^+CD8^+$ cells and minimal rearrangement of the endogenous $V\alpha$ chain that would have occurred. Selecting (C57B16; $H2^b$) stromal cells were freshly purified with minimal enzyme treatment to reduce potential loss of relevant surface molecules. The stromal cells were comprised essentially of cortical thymic nurse cells (~30%) and other epithelial cells (~30%), fibroblasts (~5%), macrophages and dendritic cells (~20%), endothelial cells (~5%), and lymphocytes (lymphocyte:stromal cell ratio of ~2:1). In these studies, multiple cell types were deliberately kept to more closely mimic the normal thymus. Current work involves separating individual cell types.

The transgenic thymocyte to adult stromal cell ratio was 5:1. A three-dimensional structure necessary to support the development of T cells was maintained by culturing the cell suspension as hanging drops. This also allows for maximum gaseous exchange and for the contact of T-cell precursors with stromal cells at the meniscus. Furthermore, as a cell suspension in 28-30 $\mu$l, the diffusion of cytokines important for cell viability and thymic selection is not hampered or too dilute. Although this system allows some three-dimensional structure to be retained, the cells were still essentially a suspension at the end of the 5-day culture. The nominal LCMV peptide (amino acids 33-41; KAVYNFATM) was usually added once at the beginning of culture at concentrations ranging from $10^{-4}$ to $10^{-14}$ M. As peptides degrade in the presence of fetal calf serum (FCS), in some experiments, the LCMV peptides were also added on a daily basis at concentrations ranging from $10^{-5}$ to $10^{-14}$ M. The P14 transgenic precursors were distinguished from the stroma-associated lymphocytes by use of Ly 5 congenic mice (for $H\text{-}2^b$ stroma) or by MHC class I haplotype (for $H\text{-}2^k$ stroma).

### *De Novo* Positive Selection of $CD4^-CD8^+$ Cells When Cocultured with Selecting MHC Stroma

The E17 transgenic ($H\text{-}2^d$) cells remained at the $CD4^+CD8^+$ stage by day 5 when cultured alone or with nonselecting MHC stroma ($H\text{-}2^k$) in the absence Figure 2A, top panel) or presence of nominal LCMV peptide (Figure 2A, lower panel) with apoptosis, as

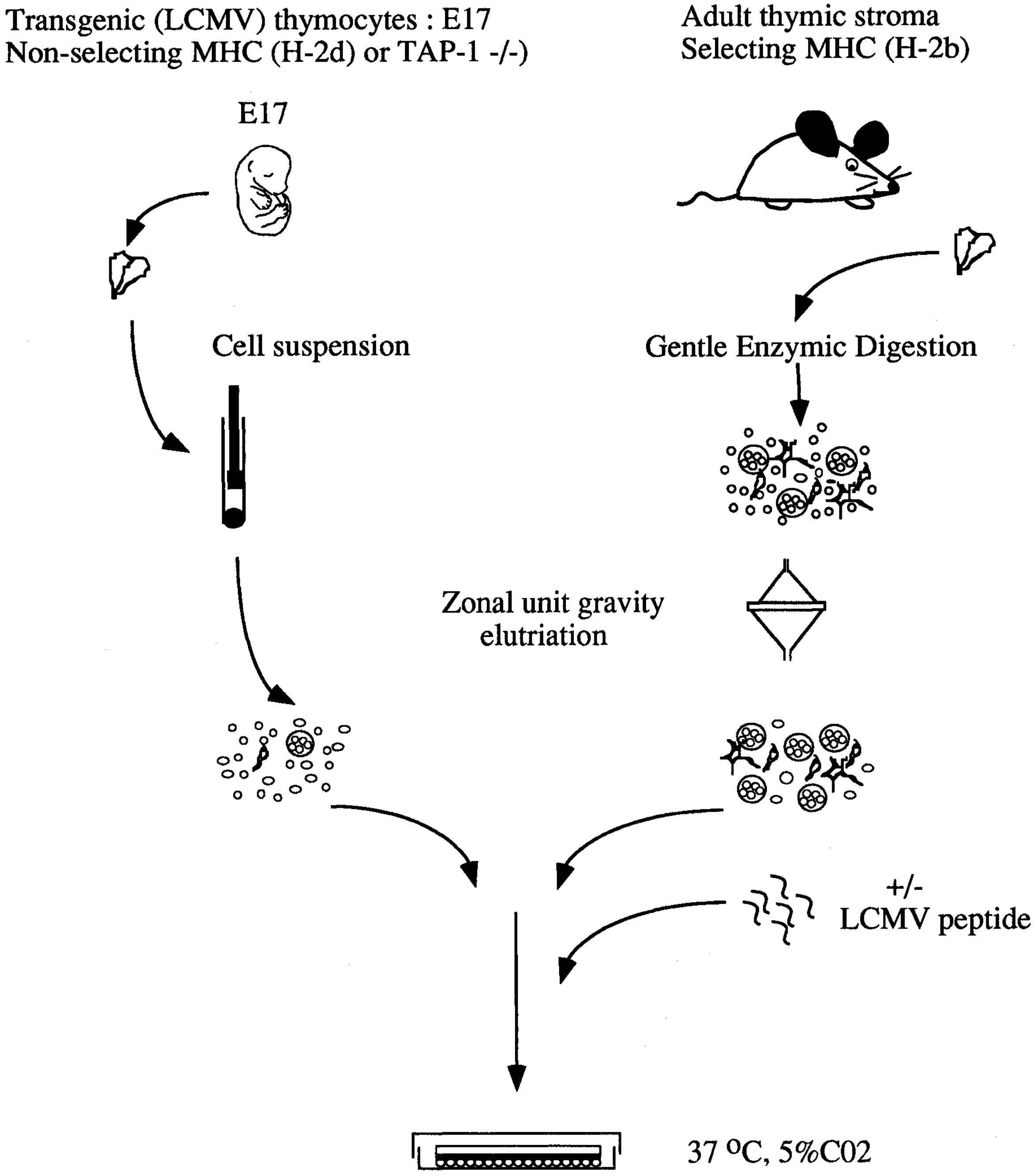

FIGURE 1 Essentially, the co-culture system involves gently digesting adult (3- to 5-week-old) H-2b thymi with enzymes then enriching for stromal cells by zonal unit-gravity elutriation. These freshly prepared stromal cells were then co-cultured in hanging drops with E17 T-cell precursors from P14 TCR transgenic mice specific for the lymphocytic choriomeningitis virus (on a nonselecting background), over a period of 4 to 5 days in the absence or presence of nominal LCMV peptide.

defined by the CD4$^{lo}$CD8$^{lo}$ phenotype (Swat et al., 1991) evident. Similarily, when cultured only with H-2$^b$ lymphocytes, the transgenic cells remained at the double-positive stage, but there was substantial apoptosis in the presence of $10^{-5}$ M peptide, consistent with previous work showing that thymocytes can self-tolerize (Pircher et al., 1992). When cultured with H-2$^b$-expressing fibroblasts (MC57), they also remained

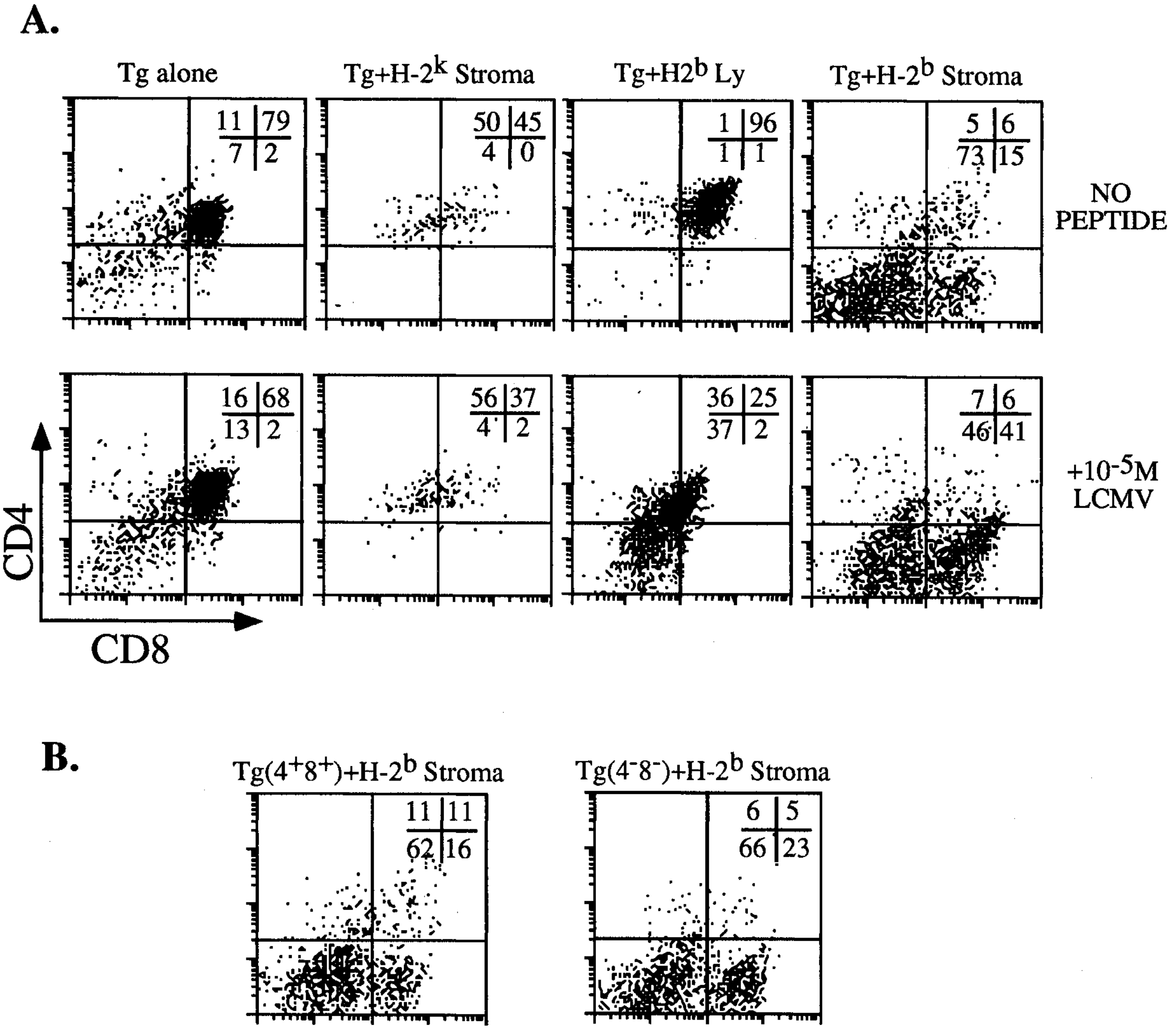

FIGURE 2 CD4$^-$CD8$^+$ cells develop when CD4$^+$CD8$^+$ P14 transgenic thymocytes are cultured in cell suspension with selecting MHC stroma but not with nonselecting MHC stroma. (A) To show that CD8 cells were not preselected, day-17 embryonic (E17) P14 transgenic thymocytes (H-2D$^b$-restricted, on a H-2$^d$ background) were cultured alone or in the presence of nonselecting (H-2$^k$) stroma. To illustrate the importance of selecting stroma in positive selection, E17 transgenic cells were co-cultured with H-2$^b$ lymphocytes, H-2$^b$-expressing fibroblasts (MC57), or selecting stroma (H-2$^b$; Ly 5.1 C57B16). These co-cultures were set up in the absence (top panel) and presence (bottom panel) of the nominal LCMV peptide (amino acids 33-41; KAVYNFATM) at a concentration of $10^{-5}$ M as described in Materials and Methods. After 5 days, cells were counted and analyzed by FACS. Transgenic cells were distinguished from stromal-related lymphocytes by Ly 5.2 or H-2$^d$ expression, then analyzed for CD4 and CD8 expression. Typical CD4 vs. CD8 profiles of transgenic cells are shown. (B) E17 transgenic cells were presorted for CD4$^+$CD8$^+$ thymocytes to eliminate the possibility that immature CD8 intermediate cells at E17 were merely expanding in culture. These CD4$^+$CD8$^+$ cells were cocultured with H-2$^b$ stroma in the absence of peptide as described and harvested on day 5. CD4 vs. CD8 profiles of recovered transgenic cells are shown. To illustrate that these cocultures can also support the differentiation of double-negative cells, CD4$^-$CD8$^-$ cells were cocultured with H-2$^b$ stroma and harvested on day 5. CD4 vs. CD8 profiles of recovered transgenic cells are shown.

$CD4^+CD8^+$, suggesting that MHC alone cannot induce positive selection (Figure 3). As a positive control, the LCMV-pulsed MC57 cells were able to activate mature T cells derived from the thymus or periphery of LCMV-transgenic H-$2^b$ mice (data not shown).

When the transgenic thymocytes were cocultured with selecting (H-$2^b$) stroma, however, significant levels of $CD4^-CD8^+$ cells developed (~15% of total cells) by day 5 (Figure 2A). This compares favorably with the *in vivo* situation; in the H-$2^b$ transgenic thymus (selecting MHC environment), approximately 18% of cells are single-positive for CD8. Given that peptides rescue positive selection in FTOC using lobes deficient in MHC class I/peptide complexes (Ashton-Rickardt et al., 1993; Hogquist et al., 1993; Sebzda et al., 1994), we examined the influence of the nominal LCMV peptide added at the onset of coculture. Positive selection was enhanced to approximately 40% in the presence of LCMV peptide at $10^{-5}$ M (Figure 2A). The reduced level of $CD4^+CD8^+$ cells in the presence of nonselecting H-$2^k$ stroma, relative to the other control cultures, is unlikely to be due to increased cell death, but rather to the presence of macrophages in the stromal preparation that we have shown avidly phagocytose presumably apoptotic $CD4^+CD8^+$ cells within 48 to 72 hr (Izon et al., 1989).

Absolute transgenic cell numbers recovered from cocultures with selecting MHC thymic stroma demonstrated at least a tenfold increase relative to cocultures with nonselecting stroma in the absence of

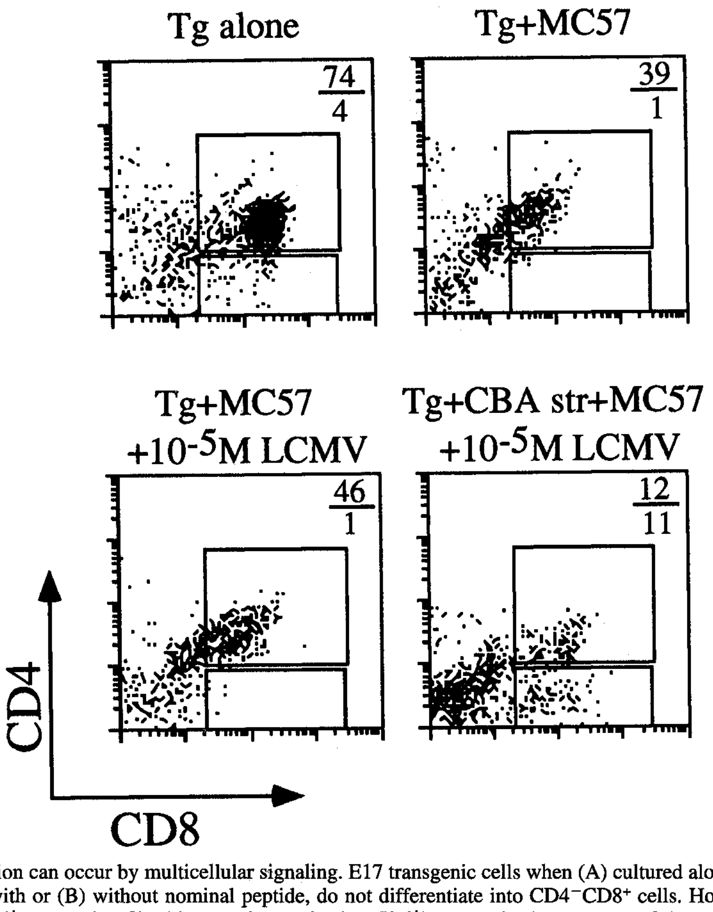

FIGURE 3 Positive selection can occur by multicellular signaling. E17 transgenic cells when (A) cultured alone or in the presence of H-$2^b$ expressing fibroblasts (C) with or (B) without nominal peptide, do not differentiate into $CD4^-CD8^+$ cells. However, when E17 transgenic cells are cocultured with H-$2^b$-expressing fibroblasts and nonselecting (H-$2^k$) stroma in the presence of the nominal LCMV peptide, some induction of $CD4^-CD8^+$ cells is evident.

TABLE I Total No.[a] of Recovered Transgenic Thymocytes (× $10^{-4}/10^6$ original E17 Tg cells)

| Co-culture | Tg alone | Tg + H-$2^k$ Str | Tg + H-$2^b$ Ly | Tg + MC57 | Tg + H-$2^b$ Str | Tg($4^+8^+$) + H-$2^b$ Str | Tg($4^-8^-$) + H-$2^b$ Str |
|---|---|---|---|---|---|---|---|
| No peptide | 1.2 ± 0.1 | 0.4 ± 0.05 | 1.5 ± 0.5 | 0.4 ± 0.1 | 4.5 ± 0.5 | 6.0 ± 0 | 7.5 ± 0 |
| $10^{-5}$ M LCMV | 1.7 ± 0.3 | 0.3 ± 0.1 | 2.5 ± 1.0 | 0.5 ± 0.1 | 7.1 ± 1.2 | | |
| $10^{-10}$ M LCMV | | | | | 6.5 ± 1.0 | | |

[a]Numbers represent mean ± S.E.M., with $n$ = 2 to 8.

TABLE II Total No.[a] of Recovered CD4$^-$CD8$^+$ Transgenic Thymocytes (× $10^{-3}/10^6$ original E17 transgenic cells)

| Co-culture | Tg alone | Tg + H-$2^k$ Str | Tg + H-$2^b$ Ly | Tg + MC57 | Tg + H-$2^b$ Str | Tg($4^+8^+$) + H-$2^b$ Str | Tg($4^-8^-$) + H-$2^b$ Str |
|---|---|---|---|---|---|---|---|
| No peptide | 0.1 ± 0.01 | 0.05 ± 0.01 | 0.15 ± 0.01 | 0.04 ± 0.01 | 11.0 ± 1.0 | 6.0 ± 0 | 7.5 ± 0 |
| $10^{-5}$ M LCMV | 0.2 ± 0.04 | 0.08 ± 0.04 | 0.25 ± 0.01 | 0.05 ± 0.01 | 18.0 ± 4.0 | | |
| $10^{-10}$ M LCMV | | | | | 39.0 ± 1.0 | | |

[a]Numbers represent mean ± S.E.M., with $n$ = 2 to 8.

peptide, and an approximately twentyfold increase in the presence of the nominal LCMV peptide (Table 1). Only approximately 1% of transgenic cells (predominantly CD4$^+$CD8$^+$ cells) were recovered after 5 days in culture in the absence of selecting stroma, compared to 6-7% in the presence of selecting stroma. Clearly, a large amount of cell loss is evident regardless of the single specificity of the transgenic TCR, most likely due to the sensitivity of immature thymocytes in culture. Despite this, the efficiency of positive selection in this model is approximately 5-10%, which compares favorably with intrathymic injections of target thymocytes (Lundberg and Shortman, 1994). Compared to transgenic cells cocultured with nonselecting stroma, there was a 200-fold increase in CD4$^-$CD8$^+$ cells recovered when transgenic cells were cocultured with selecting stroma. A similar increase was evident in the presence of $10^{-5}$ M nominal LCMV peptide; many transgenic cells would have been initially deleted due to the high concentration of peptide. In the presence of $10^{-7}$ M LCMV, there was a 300-fold increase in CD4$^-$CD8$^+$ cells, and at $10^{-10}$ M LCMV, there was 500-fold increase (Table 2).

Upregulation of the TCR as a consequence of positive selection is clearly demonstrated in Figure 4. Prior to culture, the E17 transgenic thymocytes contained virtually no TCR$^{hi}$ CD4$^-$CD8$^+$ cells, as shown by their V$\alpha$2 expression (Figure 4). Identical profiles were obtained for V$\beta$8 expression (data not shown). The E17 CD4$^+$CD8$^+$ cells and CD4$^-$CD8$^+$ intermediates in particular showed low TCR expression, in contrast to transgenic CD4$^-$CD8$^+$ cells induced when cocultured with selecting MHC stroma in the presence or absence of the nominal LCMV peptide (Figure 4).

### CD4$^-$CD8$^+$ Cells Developed *De Novo* from Presorted CD4$^+$CD8$^+$ Cells

To further establish that the CD4$^-$CD8$^+$ cells developed *de novo* and were not due to proliferation of preexisting mature cells, the E17 transgenic cells (H-$2^d$) were presorted into CD4$^+$CD8$^+$ and CD4$^-$CD8$^-$ populations prior to coculture with H-$2^b$ stroma. In both cases, significant levels (16-23%) of CD4$^-$CD8$^+$ cells developed by day 4 of culture even in the absence of nominal peptide (Figure 2B). We also examined the phenotypic and functional status of the minor subset of E17 CD4$^-$CD8$^+$ cells (4-8%) present in the nonselecting MHC thymus. In accordance with their low TCR status, these cells were immature intermediates since they were uniformly TSA-1$^{hi}$ (Godfrey et al., 1992) and HSA$^{hi}$ (Figure 5) and rapidly lost in culture—by 48 hr, 94% of the cells were CD4$^+$CD8$^+$ (Figure 6). This clearly demonstrates that CD4$^-$CD8$^+$ cells do not originate from preexisting single-positive cells and that the co-

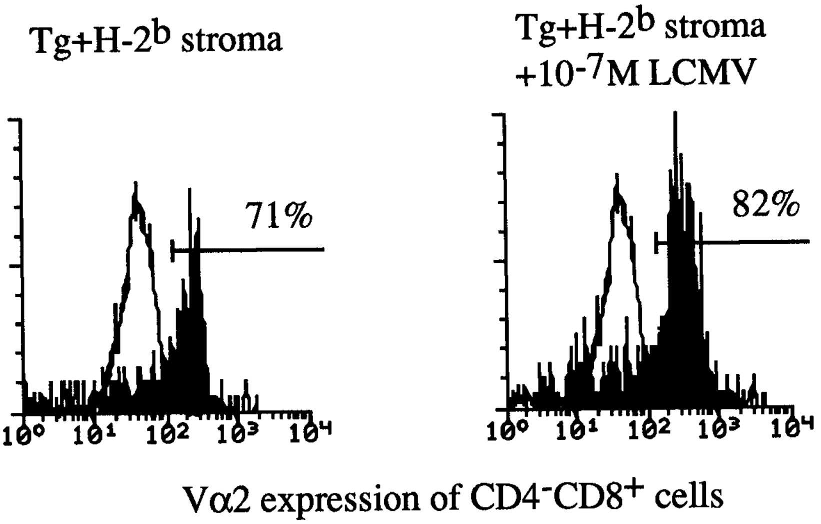

FIGURE 4 $CD4^-CD8^+$ positively selected transgenic cells have upregulated the TCR. Histograms show $V\alpha2$ expression of preculture E17 ($H\text{-}2^d$) transgenic $CD4^-CD8^+$ cells (solid line) and postcoculture $CD4^-CD8^+$ cells (shaded histogram). Transgenic cells ($H\text{-}2^d$; E17) were cocultured with $H\text{-}2^b$ stroma (Ly 5.1; C57B16) in the absence or presence of nominal LCMV peptide at a concentration of $10^{-7}$ M, for 5 days. Cells were harvested and analyzed by FACS. Transgenic cells were distinguished by Ly 5.2 expression.

culture system can support positive selection from double-negative cells presumably via $CD4^+CD8^+$ intermediates since these develop within 24 to 48 hr when the $CD4^-CD8^-$ cells are cultured alone (data not shown). Finally, if the $CD4^-CD8^+$ cells at the end of culture are simply preexisting mature cells, they would be expected to preferentially survive in cultures of transgenic thymocytes alone and even more so to proliferate in the presence of peptide-pulsed antigen-presenting cells. In neither case was this observed (Fig 6: Tg alone; Fig 3: Tg + MC57) even with irradiated $H\text{-}2^b$ splenocytes as a source of antigen-presenting cells and $10^{-5}$ or $10^{-8}$ M LCMV peptide, conditions that vigorously stimulate thymocytes and lymph nodes from LCMV transgenic $H\text{-}2^b$ mice (data not shown).

As an alternative source of transgenic thymocytes that have not received prior positive selection signals *in vivo*, we made use of P14 TCR transgenic mice on a TAP-1-deficient ($H\text{-}2^b$) background, where development of $CD4^-8^+$ but not $CD^+CD8^-$ cells is arrested (Ashton-Rickardt et al., 1993). Neonatal transgenic thymocytes from these mice were further depleted of $CD4^-CD8^+$ cells by sorting for $CD4^+$T cells (leaving only $CD4^+8^-$ and $CD4^+8^+$ cells) prior to coculturing with freshly purified thymic stroma from $H\text{-}2^b$ or $H\text{-}2^k$ mice. By day 4, $TCR^{hi}$ $CD4^-CD8^+$ cells were evident, but only with the $H\text{-}2^b$ thymic stroma, clearly demonstrating *de novo* positive selection in the $CD4^+CD8^+$ precursors, driven by endogenous or FCS-derived peptide. In these cultures, $CD4^-CD8^+$ cell numbers increased from 0 to 2-3 $\times$ $10^4$ cells per $10^6$ original transgenic cells by day 4 (data not shown).

### Positively Selected $CD8^+$ Cells Are Functionally Mature

The positively selected $CD4^-CD8^+$ cells were functionally mature both in terms of proliferation and cytotoxicity. Transgenic E17 thymocytes ($H\text{-}2^d$) were cultured alone or with selecting ($H\text{-}2^b$) or non-selecting ($H\text{-}2^d$ or $H\text{-}2^k$) thymic stroma in the absence or presence of $10^{-7}$M nominal LCMV peptide for 5 days. The stroma had been irradiated to prevent any contribution by the stroma-associated lymphocytes. After the primary coculture, the cells were harvested and further cultured for 2 days with fresh irradiated $H\text{-}2^b$ spleen cells as a source of antigen-presenting cells that had been pulsed with LCMV peptide. Transgenic cells when initially cultured alone or with nonselecting stroma for 5 days, in the absence and presence of specific peptide, showed no proliferation when challenged with fresh antigen-presenting cells loaded with LCMV peptide ($10^{-6}$M), nor were they able to specifically lyse LCMV- ($10^{-5}$M) loaded EL4 target cells. Transgenic cells cultured with selecting stroma in the absence and presence of peptide for 5 days, however, proliferated in response to LCMV- ($10^{-6}$M) loaded antigen presenting cells (Figure 7). Phenotypically, these cells remained CD4$^-$CD8$^{hi}$ and were able to specifically lyse LCMV- ($10^{-5}$M) loaded EL4 target cells (Table 3).

### CD69 Expression

The early T-cell activation marker, CD69, is transiently expressed during thymocyte selection, both positive and negative (Swat et al., 1993; Brandle et al., 1994). In the standard E17 P14 transgenic H-2d thymocyte/H-2b thymic stroma cocultures without

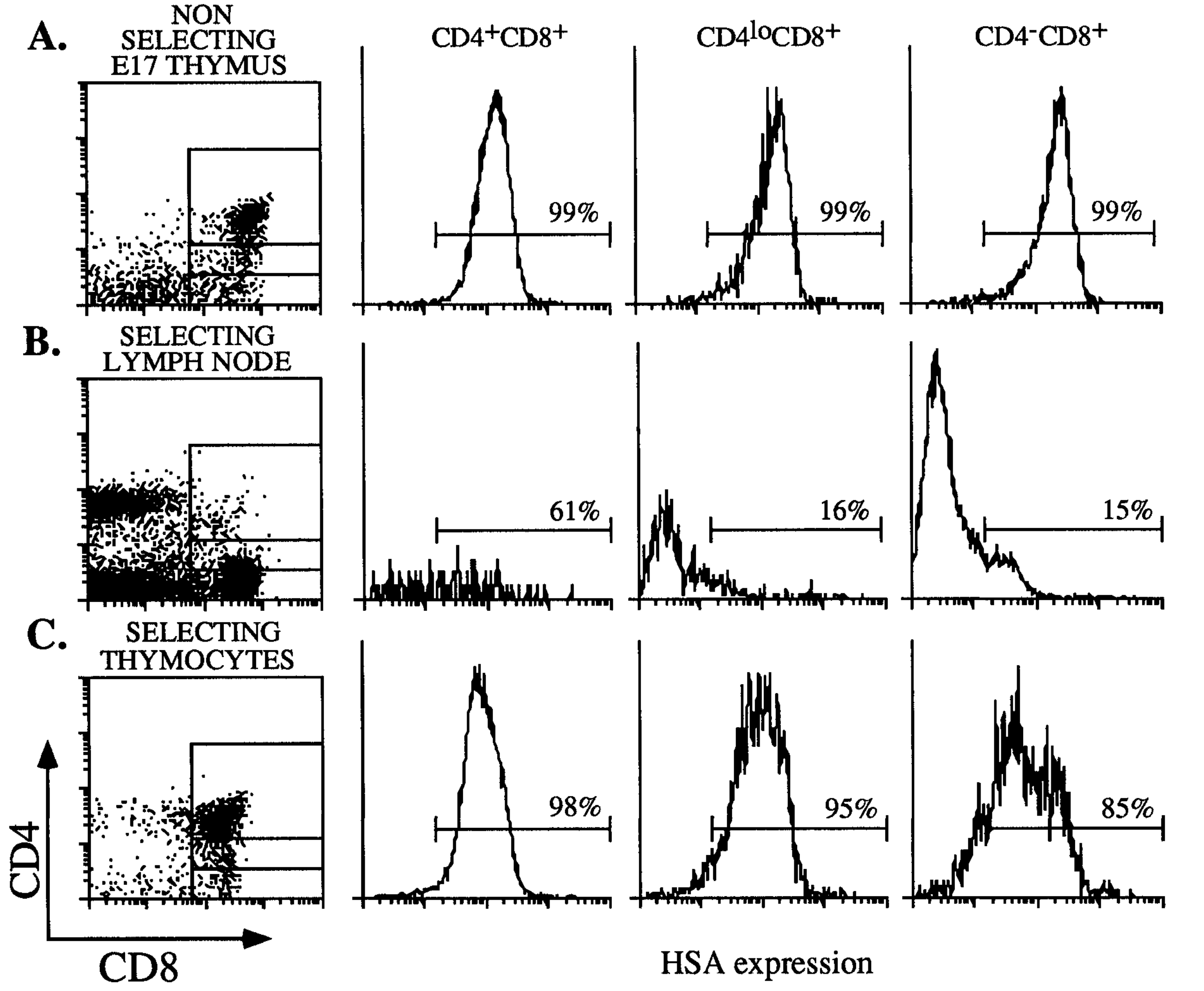

FIGURE 5 CD4$^-$CD8$^+$ cells at E17 are immature intermediates. E17 transgenic thymocytes from a nonselecting background (H-2$^d$) were analyzed for expression of HSA and comparisons made with thymocytes and lymph nodes from a transgenic mouse on a selecting background (H-2$^b$). (A) HSA expression on CD4$^+$CD8$^+$, CD4$^{lo}$CD8$^+$, and CD4$^-$CD8$^+$ subsets shows that thymocytes from a nonselecting background maintain high HSA expression in all subsets. (C) Transgenic thymocytes from a selecting background show high HSA expression on CD4$^+$CD8$^+$ cells with downregulation evident on CD4$^{lo}$CD8$^+$ and CD4$^-$CD8$^+$ cells indicating differentiation into mature T cells. (B) More complete downregulation has occurred on the fully mature T cells in the periphery.

LCMV peptide, CD69 increased from <5% at day 0, to 20-25% by day 2, reaching a maximum of approximately 50% at day 4. With $10^{-5}$ M LCMV peptide, there was a biphasic expression of CD69 (data not shown); the initial increase (day 1) coincided with the negative selection of transgenic cells with high concentration of peptide, consistent with recent findings that CD69 is also expressed on the surface of apoptosing cells (Kersh and Hedrick, 1995; Kishimoto et al., 1995). The second increase in CD69 (day 3) would be a consequence of positive selection. CD5 is another cell-surface molecule that is upregulated during positive selection (Fowlkes et al., 1985; Lanier et al., 1986). We also found CD5 was upregulated on the selected single-positive CD8 cells in the co-cultures (data not shown), providing further evidence for *de novo* positive selection.

**Positive Selection Can Involve Multiple-Cell Types**

Positive selection was not induced in the presence of nonselecting MHC stroma. To determine whether MHC alone can positively select, E17 transgenic thymocytes were cocultured with H-2D$^b$ expressing MC57 cells for 5 days. No positive selection was evident in cocultures in the absence or presence of the LCMV peptide. However, when nonselecting stroma was included with $10^{-5}$ M LCMV peptide and MC57 cells, 11% of total transgenic cells were of the CD4$^-$CD8$^+$ phenotype at day 5 (Figure 3).

### DISCUSSION

To date, thymocyte selection has been primarily studied in the embryonic thymus, but how well this

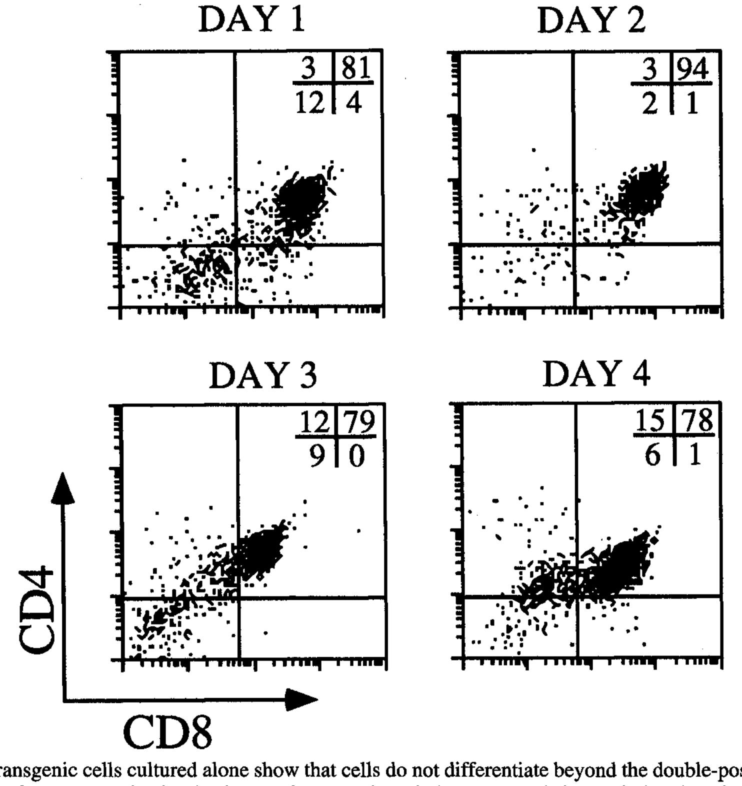

FIGURE 6 Kinetics of transgenic cells cultured alone show that cells do not differentiate beyond the double-positive stage of development. E17 transgenic thymocytes from a nonselecting background were cultured alone over a 4-day period and analyzed daily for CD4 and CD8 expression.

mimics that which occurs in the adult thymus is questionable. Here we present a cell-suspension model for *in vitro* positive selection that utilizes adult stroma, and hence will provide a good comparison with current fetal thymic models. The ease with which this system can be manipulated should enable a detailed dissection of the basis to positive selection, particularly the early kinetics and gene-activation events. Furthermore, by using adult stroma, it should allow investigations into the aetiological significance of thymic stromal-cell disorders that are manifest in the postembryonic period.

Essentially, this system involves coculturing transgenic thymocyte precursors of known TCR specificity, but in a nonselecting environment, with purified adult stroma of the selecting and nonselecting MHC type. This system has the added advantage of being able to separate out distinct stromal-cell subsets, a technique that we are currently investigating. To avoid the possibility that positive selection signals had occurred prior to culture but were not yet apparent, and to minimize the preculture handling of precursor T cells, we used E17 thymocytes from TCR transgenic mice specific to LCMV/H-$2^b$, which were maintained on a nonselecting MHC (H-$2^d$) or a TAP-1-deficient background. This is the first stage where $CD4^+CD8^+$ cells are evident; these and a low percentage of $CD3^-CD4^-CD8^+$ were $TCR^{-/lo}$, $HSA^{hi}$, and TSA-$1^{hi}$, confirming their immature status. After culture alone or in the presence of H-$2^b$-bearing nonthymic epithelial cells, the latter cells developed to $CD4^+CD8^+$ cells and there was no progression of any cells to the mature $CD4^-CD8^+$ cells, confirming that these are a consequence of *de novo* positive selection in the presence of thymic epithelium. Selecting stromal cells (H-$2^b$) were

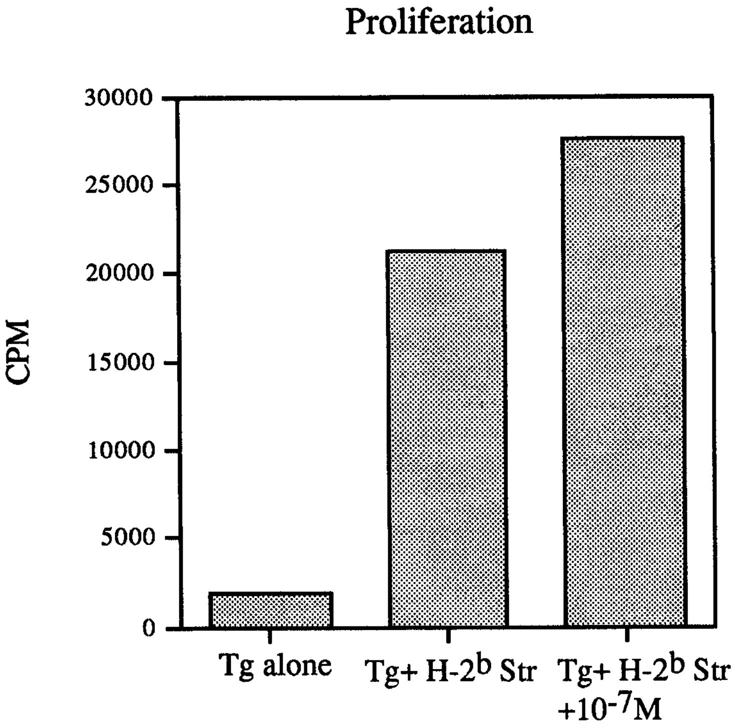

FIGURE 7 Transgenic cells selected by H-$2^b$ stroma in the absence or presence of nominal peptide are functionally mature. $CD4^-CD8^+$ cells positively selected in the absence or presence of nominal LCMV peptide ($10^{-7}$ M), proliferate in response to fresh antigen-presenting cells loaded with a higher concentration of LCMV peptide ($10^{-6}$ M). The transgenic cells when cultured alone show no such proliferation.

TABLE III Positively Selected CD4$^-$CD8$^+$ Cells Are Cytolytic[a]

| Effector:target ratio | Tg alone (EL4 + 1cmv) | Tg alone (EL4) | Tg + H-2$^b$ (EL4 + 1cmv) | Tg + H-2$^b$ (EL4) | Tg + H-2$^b$ + $10^{-7}$ M (EL4 + 1cmv) | Tg + H-2$^b$ + $10^{-7}$ M (EL4) |
|---|---|---|---|---|---|---|
| 10:1 | 0.6[b] | 0 | 71 | 3.7 | 57.2 | 5.1 |
| 3.3:1 | 2.1 | 0 | 64.5 | 2.1 | 40.3 | 3.1 |
| 1:1 | 1.4 | 0 | 62.2 | 1.1 | 26.4 | 4.3 |
| 0.4:1 | 1.7 | 0.1 | 32.7 | 1.4 | 17 | 1.9 |
| 0.1:1 | 2.5 | 0.3 | 18.2 | 0 | 4 | 2.8 |

[a]Positively selected CD4$^-$CD8$^+$ cells from cocultures containing selecting H−2$^b$ stroma, both in the absence and presence of the nominal LCMV peptide ($10^-7$ M), were able to specifically lyse target cells that were loaded with the same nominal LCMV peptide at a higher concentration ($10^{-5}$ M). Transgenic cells cultured alone under the same conditions were unable to specifically lyse LCMV-loaded target cells.
[b]% specific lysis.

freshly purified to reduce the likelihood that important surface molecules had been downregulated in culture.

It has been argued that MHC alone is all that is required to induce positive selection, with the main body of evidence being from intrathymic injections of cell lines expressing the selecting MHC into a nonselecting thymus (Hugo et al., 1992; Vukmanovic et al., 1992). These experiments, however, did not exclude the possibility that accessory signals that may be required for positive selection were received from the nonselecting thymic epithelial cells. We found that positive selection was only observed with thymic stromal cells and not when transgenic precursors were cocultured with H-2$^b$-expressing thymocytes, irradiated spleen cells, or fibroblasts (MC57) in the presence or absence of peptide. Positive selection was evident by the downregulation of the CD4 coreceptor, upregulation of TCR, and upregulation of CD69 and CD5. Similarly, Anderson and colleagues have found in reaggregate thymic organ cultures that thymic epithelium was essential for positive selection (Anderson et al., 1996). When the precursors were cocultured with H-2$^b$-expressing fibroblasts and thymic stroma from a nonselecting MHC background in the presence of peptide, we did observe some positive selection, albeit inefficient. Our data are consistent with the possibility that TCR-peptide/MHC interactions, an essential requirement for positive selection, work in conjunction with other stromal accessory signals to induce efficient complete positive selection. Certainly, although positive selection can be multicellular, it is more efficient if the correct MHC is coexpressed with appropriate differentiation molecules on dedicated thymic stroma, presumably epithelium.

The issue of peptide involvement in positive selection was briefly investigated using the nominal agonist LCMV peptide at various concentrations. Whereas positive selection did occur in the absence of specific nominal peptide, presumably due to the presence of endogenous peptide, it was enhanced in the presence of the LCMV peptide, as indicated by the increase in CD4$^-$CD8$^+$ cell numbers recovered after culture. This was most evident in cocultures containing $10^{-10}$ M LCMV. In cocultures containing higher concentrations of peptide such as $10^{-5}$ M, double-positive cells initially apoptosed, evident in kinetics studies (data not shown). By day 1, however, the double-negative cells that had converted to double-positive cells were then likely to have been selected at a concentration of peptide somewhat lower than the original level, due to degradation of the peptide in the presence of FCS. The CD4$^-$CD8$^+$ cells selected either on the endogenous peptide or in the presence of the nominal LCMV peptide were functionally mature, both in their ability to proliferate in response to LCMV-peptide-loaded antigen-presenting cells and in their capacity to specifically lyse LCMV-loaded EL4 target cells. This is significant in the light of recent publications suggesting that only antagonist peptides can positively select to produce phenotypically and functionally mature CD8$^+$ T cells.

In summary, this *in vitro* model demonstrates all the salient features of positive selection, and highlights the importance of peptide in the efficiency of selection. This system should enable a more precise dissection of the stromal cells and the role of naturally occurring self-peptides and other molecules involved in the thymic selection processes, and allow early activation events to be monitored. Furthermore, the ease with which this model can be manipulated should greatly facilitate resolution of the aetiological significance of thymic stromal cells, particularly epithelium, abnormalities that are manifest post-natally.

### MATERIALS AND METHODS

### Mice

P14 TCR transgenic mice, H-2D$^b$-restricted, were bred onto a nonselecting H-2$^d$ background and maintained at the Monash University animal house. Ly 5 congenic C57B16 mice and CBA mice were maintained at the Monash University animal house. The P14 TCR transgenic mice on a TAP-1-deficient background were a kind gift from S. Tonegawa, the Massachusetts Institute of Technology.

### Peptides

The nominal LCMV agonist peptide (amino acids 33-41; KAVYNFATM), the original cysteine at anchor position 41 in the wildtype LCMV peptide having been replaced by methionine to prevent dimer formation, were a kind gift from Hanspeter Pircher (University of Freiburg).

### T-Cell Precursors

Transgenic embryonic thymi (E17) from P14 $\alpha\beta$ TCR transgenic mice (specific to the lymphocytic choriomeningitis virus; H-2$^b$-restricted) on a H-2$^d$ (Pircher et al., 1989) or Tap-1-deficient background were the source of precursor T cells. Cell suspensions were prepared at a concentration of $3.4 \times 10^6$ cells/ml. To remove any CD8 single-positive cells, thymocytes were presorted with CD4-magnetic beads (MACS Magnetic Microbeads, Miltenyi Biotec GmbH, Bergisch Gladbach, Germany) using a VarioMACS (VarioMACS, Miltenyi Biotec GmbH, Bergisch Gladbach, Germany). Enriched cells were mostly of the CD4$^+$CD8$^+$ phenotype with a small population of CD4$^+$CD8$^-$ intermediate cells. In some experiments, pre-sorted CD4$^-$CD8$^-$ or CD4$^-$CD8$^{int}$ cell populations were also used as target cells.

### Stromal-Cell Preparation

Thymic stromal cells were prepared from Ly 5.1 congenic C57B16 mice (H-2$^b$) or CBA-CaH (H-2$^k$) mice by gentle enzymic (0.15% collagenase/0.1% DNase; Boehringer Mannheim, Germany) digestion of lymphocyte-depleted thymi. Cells were enriched to a lymphocyte to stromal-cell ratio of approximately 2:1 by zonal unit-gravity elutriation, essentially as described by Lahoud et al. (1993). Cells were subsequently resuspended in Clicks medium (GIBCO BRL, Gaithersburg, MD) that had been supplemented with 10% heat-inactivated FCS (GIBCO BRL), 2 mM L-glutamine (Flow, UK), 0.05% benzyl penicillia (CSL, Australia), 0.05% streptomycin (Sigma, St. Louis), $5 \times 10^{-5}$ M 2-mercaptoethanol (Sigma) at a concentration of $6.7 \times 10^5$ cells/ml and pulsed with peptide at the relevant concentrations.

### Co-culture

Transgenic E17 thymocytes were mixed with the stromal cells at a ratio of 5:1 and co-cultured as hanging drops in inverted Terasaki plates at 37°C, 5% $CO_2$ generally over a 5-day period. Ly5.2 expression or MHC class I (S17.71 anti H-2$^k$) distinguished P14 transgenic thymocytes from stromal-associated lymphocytes. Postculture analysis involved staining cells with Ly 5.2-FITC (PharMingen, San Diego), CD8-Biotin/Tricolour (PharMingen), and CD4-PE (PharMingen), or CD4 APC and V$\alpha$2 PE (PharMingen). Dead cells were gated out by a lymphocyte viability gate or by using propidium iodide where possible. Cells were analyzed by flow cytometry using a FACScan (Becton-Dickinson, Mountain

View, CA). Cell population gates were determined using the nontransgenic stromal-associated lymphocyte component of the coculture.

### Functional Assays

Cocultures of P14 H-$2^d$ transgenic thymocytes and freshly purified H-$2^b$ stroma were prepared and analyzed as described before with the addition that purified stroma was irradiated at 3000 rads prior to co-culture to prevent proliferation of stroma-associated lymphocytes. Cells were harvested at day 5 and placed into round-bottom wells of a 96-well plate ($5 \times 10^4$ cells/well) with IL-2 (25 units) and $5 \times 10^5$ irradiated (3000 rads) H-$2^b$ splenocytes that had been pulsed with $10^{-6}$ M LCMV peptide, for 48 hr. For the proliferation assay, ($^3$H) thymidine was added for 16 hours and incorporated radioactivity measured on a $\beta$ counter. For the cytotoxicity assay, target cells (EL4 cell line) were incubated with $^{51}$ Cr, with or without $10^{-5}$ M LCMV peptide, for 45 min. Effector cells were then incubated with target cells for 4 hr in tripling dilutions, the supernatant removed, and chromium release measured on a $\gamma$ counter. Percentage-specific lysis of target cells was calculated by:

$$\frac{\text{cpm test} - \text{cpm minimum release}}{\text{cpm maximum release} - \text{cpm minimum release}} \times 100$$

### Statistical Analysis

Viable cells were counted under a fluorescence microscope using ethidium bromide/acridine orange and standardized as the number of recovered cells per $10^6$ original E17 put into culture at day 0.

### *Acknowledgements*

We are grateful to Susumu Tonegawa for providing the P14 × Tap-1−/− mice and thank Frank Carbone for providing the MC57 cell line. This research was supported by grants from the National Health and Medical Research Council of Australia.

### *References*

Anderson G., Moore N.C., Owen J.J.T., and Jenkinson E. (1996). Cellular interactions in thymocyte development. Ann. Rev. Immunol. **14**: 73-99.

Anderson G., Owen J.J.T., Moore N.C., and Jenkinson E.J. (1994). Characteristics of an in vitro system of thymocyte positive selection. J. Immunol. **153**: 1915-1920.

Ashton-Rickardt P.G., Bandeira A., Delaney J.R., Van Kaer L., Pircher H., Zinkernagel R.M., and Tonegawa S. (1994). Evidence for a differential avidity model of T cell selection in the thymus. Cell **76**: 661-663.

Ashton-Rickardt P.G., Van Kaer L., Schumacher T.N.M., Ploegh H.L., and Tonegawa S. (1993). Peptide contributes to the specificity of positive selection of CD8+ T cells in the thymus. Cell **73**: 1041-1049.

Boyd R.L., Tucek C.L., Godfrey D.I., Izon D.J., Wilson T.J., Davidson N.J., Bean G.D., Ladyman H.M., Ritter M.A., and Hugo P. (1993). The thymic microenvironment. Immunol. Today **14**: 445-459.

Brandle D., Muller S., Muller C., Hengartner H., and Pircher H. (1994). Regulation of RAG-1 and CD69 expression in the thymus during positive and negative selection. J. Immunol. **24**: 145-151.

Brandle D., Muller C., Rulicke T., Hengartner H., and Pircher H. (1992). Engagement of the T-cell receptor during positive selection in the thymus downregulates RAG-1 expression. Proc. Natl. Acad. Sci. USA **89**: 9529-9533.

Fowlkes B.J., Edison J.L., Mathieson B.J., and Chused T.M. (1985). Early T lymphocytes: Differentiation in vivo of adult intrathymic precursor cells. J. Exp. Med. **162**: 802.

Godfrey D.I., Masciantonio M., Tucek C.L., Malin M.A., Boyd R.L., and Hugo P. (1992). TSA-1: A novel thymocyte marker discriminating immature from mature thymocyte subsets. J. Immunol. **148**: 2006-2011.

Hogquist K.A., Gavin M.A., and Bevan M. (1993). Positive selection of CD8+ T cells induced by major histocompatibility complex binding peptides in fetal thymic organ culture. J. Exp. Med. **177**: 1469-1473.

Hogquist K.A., Jameson S.C., Heath W.R., Howard J.L., Bevan M.J., and Carbone F.R. (1994). T cell receptor antagonist peptides induce positive selection. Cell **76**: 17-27.

Hugo P., Kappler J.W., Godfrey D., and Marrack P.C. (1992). A cell line that can induce thymocyte positive selection. Nature **360**: 679-682.

Izon D.J., Boyd R.L., Waanders G.A., and Kelso A. (1989). The myelopoietic inducing potential of mouse thymic stromal cells. Cell. Immunol. **124**: 264-277.

Kersh G.J., and Hedrick S.M. (1995). Role of TCR specificity in CD4 versus CD8 lineage commitment. J. Immunol. **154**: 1057-1068.

Kishimoto H., Surh D.C., and Sprent J. (1995). Upregulation of surface markers on dying thymocytes. J. Exp. Med. **181**: 649-655.

Kouskoff V., Vonesch J., Benoist C., and Mathis D. (1995). The influence of positive selection on RAG expression in thymocytes. Eur. J. Immunol. **25**: 54-58.

Lahoud M., Vremec D., Boyd R.L., and Shortman K. (1993). Characterization of thymic nurse-cell lymphocytes, using an improved procedure for nurse-cell isolation. Dev. Immunol. **3**: 103-112.

Lanier L.L., Allison J.P., and Phillips J.H. (1986). Correlation of cell surface antigen expression on human thymocytes by multicolor flow cytometric analysis: Implications for differentiation. J. Immunol. **137**: 2501.

Lundberg K., and Shortman K. (1994). Small cortical thymocytes are subject to positive selection. J. Exp. Med. **179**: 1475-1483.

Merkenschlager M., and Fisher A.G. (1994). In vitro construction of graded thymus chimeras. J. Immunol. Meth. **171**: 177-188.

Pawlowski T., Elliot J.D., Loh D.Y., and Stearz U.D. (1993). Positive selection of T lymphocytes on fibroblasts. Nature **364**: 642-645.

Pircher H., Burki K., Lang R., Hengartner H., and Zinkernagel R.M. (1989). Tolerance induction in double specific T-cell receptor transgenic mice varies with antigen. Nature **342**: 559-561.

Pircher H., Muller K., Kyewski B.A., and Hengartner H. (1992). Thymocytes can tolerize thymocytes by clonal deletion *in vitro*. Int. Immunol. **4**: 1065-1069.

Sebzda E., Wallace V.A., Mayer J., Yeung R.S.M., Mak T.W., and Ohashi P.S. (1994). Positive and negative thymocyte selection induced by different concentrations of a single peptide. Science **263**: 1615-1618.

Swat W., Dessing M., von Boehmer H., and Kisielow P. (1993). CD69 expression during selection and maturation of CD4+8+ thymocytes. Eur. J. Immunol. **23**: 739-746.

Swat W., Ignatowicz L., von Boehmer H., and Kisielow P. (1991). Clonal deletion of immature CD4+8+ thymocytes in suspension culture by extrathymic antigen-presenting cells. Nature **351**: 150-153.

Vukmanovic S., Grandea A.G., Faas S.J., Knowles B.B., and Bevan M.J. (1992). Positive selection of T lymphocytes induced by intrathymic injection of a thymic epithelial cell line. Nature **359**: 729-732.

